# Observations on the Influenza

**Published:** 1803-09-01

**Authors:** T. Peaal

**Affiliations:** Aberdeen


### Observations on the Influenza; *communicated by* Mr. T. Peaal, *of Aberdeen*.

THE Influenza, or epidemic catarrhal fever, appeared first in Aberdeen towards the latter end of February; it seized those who were affected with it in the same manner as the continued fever, which generally prevails here, described by Dr. Ferguson in his Essay on Fever. It could be easily distinguished from it by the patient not being so violently affected, and by the disorder of the lungs and catarrhal spit. The patients complained of a violent pain in their head, accompanied with languor and soreness of their bones. In some, the affection of the lungs was very violent, attended with pain in the breast, cough, dyspnœa, and catarrhal spit, not unlike pneumonia; while in others, the cough, expectoration, &c. were but slight. The pulse was small and quick in most of the cases. A great many, who were affected with influenza, did not apply for medical advice; with them, for a considerable time, it went by the name of a cold, and was not considered as a species of catarrhal fever until it appeared as an epidemic in other places. It is a general custom with many in Aberdeen and its vicinity to bleed in the month of March, whether they are ill or not, with a view (as I suppose) to prevent inflammatory disorders, which generally ensue in the spring after hard work. I made it an object of inquiry to ask whether those who were bled in this month had the disease milder, or escaped it altogether; so satisfactory was my inquiry in this particular, that all who were bled, and not previously affected with influenza, generally escaped it. I bled about fifty, and nearly the same number affected with influenza applied to me for medical advice, who had not been bled. The greater part of these patients were men, and none under ten years of age. I bled the most of them, and found it very servicable in moderating all the symptoms. I was called about this time to some young children in fever, but could not distinguish whether it was influenza or fever from cold. Towards the latter end of March, the influenza was at its height. Vomits were generally applied by the patients themselves. In some cases, where the pain in the head and fever were violent, temporal blisters and the saline mixture were had recourse to. Where the affection of the lungs was severe, a fomentation of warm vinegar and spirits applied to the pained part, at the same time using the mucilaginous mixture with tincture of of opium internally, seldom failed to make the patient soon better. The influenza was remarkably mild, and after the third or fourth day, the patients generally felt an alleviation of all the symptoms, excepting in cases where it was combined with chronic cough and catarrh. Catarrh is very common in Aberdeen, and not unfrequently chronic, returning and affecting those of a weakly habit who are much exposed to the coldness of the atmospheric air; it is to be distinguished from influenza, by its not being contagious, but generally proceeding from atmospheric influence, and by its duration. The present and former epidemic influenzas would appear to be very infectious, from the number so suddenly affected, from the rapidity of its propagation, and from its preserving its form amidst the different states of temperature, climate, and different habits of life.

Most of the epidemic exanthemata are infectious, and affect persons but once in their lives. Query, were those affected with the influenza, when it raged last in 1788, exempted from it at this time? I was for three or four days very ill with it in 1788, but have escaped the late epidemic. All those who have applied to me at this time, were not affected with the influenza when it prevailed last. Those who were violently affected, continued to have a little expectoration in the morning, after all the other symptoms were gone; the weather becoming warm, has tended much to restore the mucous glands to their healthy state.

The measles are the present epidemic in Aberdeen, they are affecting a great many children, and seem to be more violent than the influenza. The fever attending them is particularly severe. Bleeding with the lancet or leeches; gentle laxatives, and mucilaginous drinks, are the common agents employed in the cure.

